# Conventional breeding, marker-assisted selection, genomic selection and inbreeding in clonally propagated crops: a case study for cassava

**DOI:** 10.1007/s00122-015-2555-4

**Published:** 2015-06-21

**Authors:** Hernán Ceballos, Robert S. Kawuki, Vernon E. Gracen, G. Craig Yencho, Clair H. Hershey

**Affiliations:** International Center for Tropical Agriculture (CIAT), Apartado Aéreo, 6713 Cali, Colombia; National Crops Resources Research Institute (NaCRRI), Kampala, Uganda; West African Center for Crop Improvement (WACCI), Cornell University, Accra, Ghana; Department of Horticultural Science, North Carolina State University, Raleigh, USA

## Abstract

**Key message:**

**Consolidates relevant molecular and phenotypic information on cassava to demonstrate relevance of heterosis, and alternatives to exploit it by integrating different tools. Ideas are useful to other asexually reproduced crops.**

**Abstract:**

Asexually propagated crops offer the advantage that all genetic effects can be exploited in farmers’ production fields. However, non-additive effects complicate selection because, while influencing the performance of the materials under evaluation, they cannot be transmitted efficiently to the following cycle of selection. Cassava can be used as a model crop for asexually propagated crops because of its diploid nature and the absence of (known) incompatibility effects. New technologies such as genomic selection (GS), use of inbred progenitors based on doubled haploids and induction of flowering can be employed for accelerating genetic gains in cassava. Available information suggests that heterosis, non-additive genetic effects and within-family variation are relatively large for complex traits such as fresh root yield, moderate for dry matter or starch content in the roots, and low for defensive traits (pest and disease resistance) and plant architecture. The present article considers the potential impact of different technologies for maximizing gains for key traits in cassava, and highlights the advantages of integrating them. Exploiting heterosis would be optimized through the implementation of reciprocal recurrent selection. The advantages of using inbred progenitors would allow shifting the current cassava phenotypic recurrent selection method into line improvement, which in turn would allow designing outstanding hybrids rather than finding them by trial and error.

## Introduction

Cassava (*Manihot esculenta* Crantz) is a perennial shrub originated in the neotropics. Its most important product is the starchy roots used as a source of caloric energy by millions of people, particularly in Sub-Saharan Africa. It is also a competitive source of starch; cassava is the second most important source of starch worldwide, after maize (Stapleton 2012; Norton 2014), and is the starch most traded internationally. Dried cassava root chips are also used at industrial levels for animal feeding and ethanol production. Cassava is typically propagated through the use of stem cuttings. Since nearly all known landraces and bred varieties are directly derived from a cross between two heterozygous parents, the plants that farmers grow are clonally propagated hybrids. As such, cassava can be used as a model for clonal crops with the advantage that it is grown annually and does not have the complication of polyploidy that several other clonally propagated species have, e.g., potato, sweetpotato, yam and banana. Although Magoon et al. suggested in 1969 that certain portions of the genome may be duplicated; cassava should be considered as a functional diploid species (Wang et al. 2011; Westwood 1990). This article aims at evaluating the potential effectiveness of different technologies available for the genetic improvement of cassava and, by extension, for other clonal crops.

Cassava breeding is based on the production of full-sib and/or half-sib progenies which are then evaluated through phenotypic mass selection (Jennings and Hershey 1985; Jennings and Iglesias 2002; Ceballos et al. 2012). Little or, more often than not, no attention is paid to family structure in the selection process. Breeders focus their attention on evaluating and selecting individual genotypes regardless of the family origin. It is these individual selected genotypes that will eventually be released as varieties by breeders and grown by farmers. A key feature of this process is that all genetic effects (additive, dominant, over-dominant and epistatic) not only influence the breeder’s decisions, but also can be exploited in the cloned genotypes grown by farmers. The clonal reproduction of cassava allows multiplication of individual genotypes in such a way that environmental and genetic factors affecting their performance can be separated. This is important because within-family genetic effects can be properly estimated in cassava.

An important and distinctive characteristic of cassava breeding is that it uses heterozygous progenitors to produce the varieties grown by farmers: clonally propagated hybrids. This fact places cassava in a unique position compared with autogamous or commercial hybrid crops (such as maize, sorghum and sunflower) whose breeding is based on the use of homozygous progenitors. Breeding of many other clonal crops is also based on heterozygous progenitors. This distinction based on level of heterozygocity of parents is important, since the partitioning of the genetic variances differs drastically from families derived from fully or partially homozygous lines, as shown in Table 1. In the typical cassava breeding process, as stated above, all genetic effects are exploited but they are asymmetrically distributed in the between- and within-family components. Half the additive variance ($\sigma_{\text{A}}^{ 2}$) present in the parental generation will express as differences between the full-sib families generated. The remaining half will be contained in the within-family variation. On the other hand, only ¼ of the dominance variance ($\sigma_{\text{D}}^{ 2}$) is contained in the between-family variation whereas the remaining ¾ is in the within-family component. For these estimates to be valid, all models assume the absence of epistatic effects. However, as demonstrated below, for complex traits such as fresh root yield (FRY), epistasis has been proven to be significant. As is the case with $\sigma_{\text{D}}^{ 2}$, a considerable portion of epistatic effects will be expressed in the within-family variation.

**Table 1:** Partitioning of additive ($\sigma_{\text{A}}^{ 2}$) and dominance ($\sigma_{\text{D}}^{ 2}$) genetic effects in different types of families (Hallauer and Miranda Fo 1981; Venvcosky and Barriga 1992; Kearsey and Pooni 1996)

Type of family	Inbreeding coefficient	Between families		Within families		Total	
		$\sigma_{\text{A}}^{ 2}$	$\sigma_{\text{D}}^{ 2}$	$\sigma_{\text{A}}^{ 2}$	$\sigma_{\text{D}}^{ 2}$	$\sigma_{\text{A}}^{ 2}$	$\sigma_{\text{D}}^{ 2}$
HS	0	1/4	0	3/4	1	1	1
FS	0	1/2	1/4	1/2	3/4	1	1
S_1_/F_3_	1/2	1	1/4	1/2	1/2	3/2	3/4
S_2_/F_4_	3/4	3/2	3/16	1/4	1/4	7/4	7/16
S_3_/F_5_	7/8	7/4	7/64	1/8	1/8	15/8	15/64
……..							
S_∞_/F_∞_	1	2	0	0	0	2	0

A notable feature of the information provided in Table 1 is what happens when varying degrees of inbreeding are used. There is a clear, gradual and consistent reduction in the relevance of $\sigma_{\text{D}}^{ 2}$, which is proportional to the increase in the magnitude of $\sigma_{\text{A}}^{ 2}$, as inbreeding takes place. With fully homozygous inbred genotypes, all the genetic variation lies in the between-lines component, since there is no longer genetic variation within the inbred line. Moreover, differences among inbred lines work as a *magnifying glass* on the additive effects present in the parental population (as they will be actually estimating $2\sigma_{\text{A}}^{ 2}$). There is no measurable $\sigma_{\text{D}}^{ 2}$ in inbred lines as intra-locus interactions have been eliminated through the inbreeding process. Epistatic effects (inter-loci interactions) are seldom properly considered but it is important to acknowledge that they are still present and will influence the differences between inbred lines.

## Genetic variances and their effects in cassava

Although there are many research articles describing QTLs and molecular maps in cassava, there is relatively little information regarding Mendelian and quantitative genetics. For example, in spite of four decades of breeding for resistance to cassava mosaic disease (CMD), perhaps the most studied disease in this crop, its mode of inheritance is still not clear, nor is it clear if there are just one or several different sources of resistance. Fortunately, however, this lack of knowledge is gradually being overcome in support of the long held hypothesis of major effect of (at least) monogenic resistance (Rabbi et al. 2014).

Three different diallel crosses in cassava were developed and subsequently tested in three different environmental conditions in Colombia (Cach et al. 2005, 2006; Calle et al. 2005; Jaramillo et al. 2005; Pérez et al. 2005a, 2005b). These studies included quantification of the between- ($\sigma_{\text{B}}^{ 2}$) and within-family ($\sigma_{\text{W}}^{ 2}$) genetic variation, which in turn allowed testing the significance of epistasis, in addition to the usual general (GCA) and specific (SCA) combining ability effects. Table 2 provides a summary of the results of these studies for FRY and dry matter content (DMC). Within-family is considerably larger than the between-family variances, particularly for FRY. This finding is relevant because it is the within-family variation that most closely resembles the selection unit used by most cassava breeders, i.e., the individual genotypes within families (with little or no regard to the family performances).

**Table 2:** Variance estimates (standard errors within parenthesis) for FRY and DMC in three different diallel sets evaluated in the three environments for cassava production in Colombia

Genetic parameter	Fresh root yield (t ha^−1^)			Dry matter content (%)		
	Acid soil	Sub-humid	Mid-altitude	Acid soil	Sub-humid	Mid-altitude
$\sigma_{\text{G}}^{ 2}$ (between)	1.65	13.09	42.78	1.60	0.77	0.35
	(2.95)	(4.74)	(13.27)	(0.66)	(0.29)	(0.12)
$\sigma_{\text{G}}^{ 2}$ (within)	21.08	127.21	288.93	3.22	5.56	0.12
	(2.30)	(7.65)	(1918)	(0.17)	(0.31)	(0.12)
$\sigma_{\text{A}}^{ 2}$	−1.49	17.82	11.88	3.38	1.45	0.99
	(6.32)	(13.75)	(24.67)	(2.40)	(0.99)	(0.47)
$\sigma_{\text{D}}^{ 2}$	9.03	23.87	152.11	0.87	0.77	−0.21
	(7.93)	(11.15)	(49.08)	(0.67)	(0.50)	(0.13)
Epistasis test	15.05	100.40	168.91	0.87	4.26	−0.32
	(6.74)	(12.74)	(39.72)	(1.29)	(0.67)	(0.92)

In general, it is considered that DMC is relatively easier to improve (higher realized heritability) than FRY (Kawano et al. 1998). Results in Table 2 tend to support this concept. Relatively speaking, $\sigma_{\text{W}}^{ 2}$ is much larger for FRY than for DMC, when compared with $\sigma_{\text{B}}^{ 2}$. In other words, predictions based on GCA would be more reliable for DMC than for FRY. This is in fact the case as the estimates of $\sigma_{\text{A}}^{ 2}$ for DMC are much larger than those for $\sigma_{\text{D}}^{ 2}$, whereas the opposite is the case for FRY. Finally, the tests for epistasis using diallel crosses also suggest a similar trend with strong epistatic effects for FRY, whereas for DMC the test reached statistical significance only for the sub-humid environment. Results from Table 2 agree with the realized gains for these two traits, reported by Kawano et al. (1998). Wide genetic variability for DMC has been reported for Africa (Kawuki et al. 2011a), as well as the suggestion that breeding for it is efficient.

In cassava, resistance to pests and diseases such as super-elongation disease, or reaction to whiteflies or thrips, are considered to have high heritability. For example, the resistance to the trips *Frankliniella williamsi* depends on the pubescence on the leaves in the apical shoot which is stable and readily identifiable. Resistance to the whitefly *Aleurotrachelus socialis* is linked to antibiosis (Bellotti 2002). An interesting example in this regard is the resistance against *A. socialis* found in a landrace from Ecuador (MEcu 72). For this trait, GCA and SCA mean squares were, respectively, 130.1 (significant at *P* < 0.01) and 6.3 (not significant). The epistasis test was also not significant (Perez et al. 2005b). Even where a single dominant gene controls the resistance to a given pest or disease, the effect will show as very significant GCA or $\sigma_{\text{A}}^{ 2}$. This is because the dominant resistance will express in most of the progenies from the resistant progenitor (as was the case with MEcu 72), with relatively little relevance of SCA effects or $\sigma_{\text{D}}^{ 2}$.

Several studies (many of them conducted in Africa) allow the quantification of the relative importance of GCA and SCA in different cassava traits. Table 3 summarizes the results of these studies grouped as diallel analyses and North Carolina II designs (Bueno 1991; Chipeta et al. 2013; Easwari Amma et al. 1995; Kamau et al. 2010; Lokko et al. 2006; Njenga et al. 2014; Owolade et al. 2006; Parkes et al. 2013; Were et al. 2012; Zacarias and Labuschagne 2010). Because of the different designs of these studies (fixed versus random genetic effects), the summary provided in Table 3 uses the reported mean squares for GCA and SCA to estimate their ratio (GCA/SCA). The table illustrates the relative importance of additive and non-additive genetic effects in different types of traits that are economically important for cassava.

**Table 3:** Relative importance of GCA and SCA mean squares (expressed as the GCA/SCA ratio) evaluated in different genetic designs for different relevant traits in cassava

First author	FRY	DMC	HI	PT	CMD**	CBSD**	CMB**	CGM**
Bueno (1991)*	0.49							
Cach	5.0	8.4	3.5	5.9				
Calle	3.5	17.3	6.1	19.7				
Chipeta	1.0						0.7	1.9
Easwari	2.1	0.2	1.1					
Jaramillo	2.3	5.8	7.0	1.6				
Kamau	1.1	1.3	0.8		1.1			1.1
Kulembeka	1.1		3.3			21.5		
Lokko					9.0			
Njenga	0.8							
Parkes	0.5	1.6	1.0		1.7			
Were	0.4				12.0			
Zacarias	0.2		3.5			0.8	2.7	
Mean	1.6	5.8	3.3	9.1	5.9	11.2	1.7	1.5

*FRY* fresh root yield, *DMC* dry matter content, *HI* harvest index, *PT* plant type, *CMD* cassava mosaic disease, *CBSD* cassava brown streak disease, *CMB* cassava mealybug, *CGM* cassava green mite

* For this author, the ratio presented is $\sigma_{\text{A}}^{ 2}$ / $\sigma_{\text{D}}^{ 2}$

** Based on reactions on 1–5 scale, where 1 = least damage and 5 = most damage

In addition to the information presented in Table 3, non-additive genetic effects have been reported for carotenoids content in the root (Akinwale et al. 2010). The averages provided at the bottom of Table 3 should be interpreted with caution, since information for different traits relies on different studies. They are useful, however, to explore indications of the relative importance of GCA and SCA for different traits. As stated above, for complex traits such as FRY, SCA plays an important role as the ratio is slightly above one. In many individual studies, however, the ratio is below one, indicating relatively larger relevance of SCA compared with GCA. For variables that are considered to have a relatively simpler inheritance, the GCA/SCA ratio increases sharply. For plant architecture-related traits such as harvest index (HI) and plant type (PT), the mean GCA/SCA ratios were, respectively, 3.3 and 9.1. For DMC, the ratio was also much higher than one (5.8). The magnitudes vary widely for resistance to different viral diseases. In the case of CMD, the ratio ranged from 1.1 to 12.0 with an average of 5.9. For cassava brown streak disease (CBSD), there were two very contrasting reports, with GCA/SCA ratios ranging from 0.8 to 21.5. In the case of resistance to arthropod pests, there has been more than one report in the literature only for cassava mealy bug (CMB) and cassava green mite (CGM). GCA/SCA ratios in these latter two cases were relatively low (1.7 and 1.5, respectively). However, for these two pests, the control strategy relies heavily on biological control rather than genetic resistance per se. While there can be a good level of confidence in heritable resistance to CGM, resistance to CMB is much more tentative.

## Inbreeding depression and heterosis in cassava

Heterosis in crops is linked to non-additive genetic effects (dominance, over-dominance and epistasis). Cross-pollinated crops such as maize and cassava demonstrate large heterotic effects, whereas self-pollinated crops such as soybean, rice or wheat were historically believed to show little or no heterosis. However, hybrid rice varieties have been found to yield about 15–20 % more than even the best of the improved high yielding inbred varieties. In China, hybrids account for more than half of all area under rice cultivation in the country as of 2010 (Spielman et al. 2013). The unexpected impact of hybrid rice highlights the relevance of non-additive genetic effects even for crops that were historically considered to express a relatively low level of heterosis.

Generally, crops that show strong heterotic effects also have important inbreeding depression. In fact, it has been suggested that heterosis and inbreeding depression are, to some extent, opposite sides of basically the same phenomenon (Miranda Fo 1999). In other words, the same factors that increase performance in the heterozygous hybrid are related to inbreeding depression in the homozygous progenitors. One could perceive inbreeding depression and heterosis to be opposites for dominance and over-dominance effects but not necessarily for epistasis. It is expected, therefore, that traits showing strong heterosis (non-additive effects) should also be subjected to strong inbreeding depression. Similarly when traits are mostly additive in nature, inbreeding depression should be negligible. Rojas-C et al. (2009 ) provide an analysis of inbreeding depression in eight S_1_ populations derived, respectively, from eight elite hybrid cassava cultivars. As expected, inbreeding depression for FRY was very high with an average of 63.9 % (Table 4), whereas inbreeding depression for DMC was considerably lower (average of 5.3 %) because of additive genetic effects probably playing a major role.

**Table 4:** Inbreeding depression (ID) as percentage (%) of the performance from the S_0_ generation, measured in eight S_1_ cassava families (adapted from Rojas et al. 2009)

Family	Plant height (cm)		Root yield (kg/plant)		Foliage yield (kg/plant)		Harvest index (0–1)		DMC (%)	
	S_0_	ID	S_0_	ID	S_0_	ID	S_0_	ID	S_0_	ID
Average	215	10.1	4.4	63.9	2.3	37.9	0.62	26.5	31.3	5.3
Range*	Max	Min	Max	Min	Max	Min	Max	Min	Max	Min
	38.2	−19.5	95.8	−23.1	85.2	−43.5	74.8	−15.9	26.4	−15.2

* Maximum and minimum quantification of inbreeding depression observed among the eight S_1_ families

A similar study using six S_1_ families was reported in 2011 (Kawuki et al. 2011b). Inbreeding depression for FRY was 61.2 %, which is remarkably similar to the values presented in Table 4. In the case of DMC, these authors reported slightly higher values for inbreeding depression (12.9 %) compared with those by Rojas-C et al. (2009). On the other hand, Kawano (CIAT Annual Report 1974) reported similar inbreeding depression for root and foliage yield, and HI was not affected.

## Summary of available information on the inheritance of different traits in cassava

In general, therefore, it can be hypothesized that the more complex the trait is, the higher the relevance of within-family variation, and non-additive genetic effects such as dominance and epistasis. This is the case of FRY. Traits that are considered easier to improve show relatively lower importance of the within-family variation and consistently higher relevance of additive genetic effects (e.g., GCA). This is the case for DMC, HI and PT. For resistance to CMD and CBSD, the average GCA/SCA ratios were 5.9 and 11.2, respectively, but individual reports vary widely. For many other pests and diseases (e.g., whiteflies, thrips, cassava bacterial blight, super-elongation disease), there is still very limited information to be able to draw reliable conclusions. Further studies on the inheritance of these important traits are needed.

Results presented above agree with those of the much better studied maize. Grain yield showed the largest relative importance of non-additive genetic effects among 19 traits analyzed (Hallauer and Miranda Fo 1981, p. 116). Within-family variation was also considerably more relevant for grain yield than for traits such as ear length or ear height (Hallauer and Miranda Fo 1981, p. 253). Epistasis has also been found to be relevant for grain yield (Lamkey et al. 1995; Wolf and Hallauer 1997; Crow 2000 among many more reports in the literature). As in the case of cassava, additive and dominance genetic effects explain a great proportion of genetic variation in corn. Performance of the best hybrids, therefore, depends mainly on additive and dominance variances, but gets an extra boost from epistasis. In other words, what distinguishes the success of best commercial hybrids from the rest is the extra bit of genetic superiority derived from epistatic effects (Crow 2000). Similar conclusions are mentioned by Hallauer and Miranda Fo in 1981 (p. 294) and Mikel and Dudley (2006). Moreover, even in tree breeding programs, similar conclusions have been reached. Zhao et al. (2014) reported that SCA values were higher than GCA for relevant traits in *Betula platyphylla.*

## Application of molecular technologies in cassava research

Molecular biology has a large spectrum of applications in agriculture and there have been several examples of successful applications in cassava. Molecular diagnostics can be used for diseases of complex etiology, such as frogskin disease (Alvarez et al. 2009; Calvert et al. 2008); detection and quantification of viral diseases (Monger et al. 2001; Hernández Pérez et al. 1999; Kaweesi et al. 2014) and analysis of their genetic diversity (Calvert et al. 2008; Legg et al. 2011; Monger et al. 2001); in the characterization and diversity studies of fungal and bacterial diseases (Restrepo and Verdier 1997; Álvarez and Molina 2000; Álvarez et al. 2003), as well as in gene expression studies in host–pathogen interactions (Kemp et al. 2004, 2005; Fregene et al. 2004; Hong and Stanley 1995; Maruthi et al. 2014). Molecular markers have been fundamental for the identification and introgression of resistance to CMD in Latin American germplasm (Egesi et al. 2006; Fregene et al. 2000; Okogbenin et al. 2007). An interesting application of molecular markers has been for the dissection of the pathway leading to post-harvest physiological deterioration (PPD) in cassava roots (Reilly et al. 2007).

Another important application of molecular biology for cassava is in diversity studies (Asante and Offei 2003; Beeching et al. 1993; Castelo Branco and Schaal 2001; Colombo et al. 2000; Hurtado et al. 2008; Kawuki et al. 2009; Roa et al. 1997); ethnobotany, evolutionary and hybridization studies (da Silva et al. 2003; Duputié et al. 2007; Elias et al. 2001; Pujol et al. 2005; Roa et al. 2000; Salik et al. 1997) and basic research on the origin of cultivated cassava and its taxonomy (Duputié et al. 2011; Olsen 2002, 2004; Second et al. 1997; Olsen and Schaal 1999, 2001). Genetic identity of cassava clones cultivated by farmers through the use of molecular markers has been also reported (Alzate-G et al. 2010).

However, without doubt, one of the most important applications of molecular marker technology in relation to genetic improvement of cassava involves the use of marker-assisted selection (MAS). The use of MAS in plant breeding has continued to grow in the public and private sectors (Heffner et al. 2009). As summarized by Xu and Crouch in 2008, there are four main applications of molecular markers technologies in crop breeding:

(i) Traits difficult to improve through conventional phenotypic selection—because they are expensive or time-consuming to measure, or have low penetrance or complex inheritance. An example in cassava would be the frustrating work on PPD which is prone to large experimental errors and requires a large number of roots (García et al. 2013).
(ii) Traits whose selection depends on specific environments or developmental stages for expression of the target phenotype. The best example (perhaps the only case where MAS has been applied so far in cassava genetic improvement) was the selection for resistance to CMD in Colombia, a country where this viral disease is fortunately absent (Akano et al. 2002; Fregene et al. 2000; Blair et al. 2007). Other examples of possible application are selection for resistance to whiteflies or cassava frogskin disease at the experimental station, because these two biotic problems are actively excluded for phytosanitary concerns.
(iii) Maintenance of recessive alleles during backcrossing or for speeding up backcross breeding in general. However, this application is not feasible in cassava because of the lack of homozygous recurrent parents for backcrossing. Although selection for haplotype blocks could be considered, the complete recovery of the recurrent parent would never be possible.
(iv) Pyramiding multiple monogenic traits (such as pest and disease resistances or quality traits) or several QTL for a single target trait with complex inheritance (such as drought tolerance or other adaptive traits). This is also a very useful application of molecular markers; however, markers associated with multiple sources of resistance have not been available up to now. Early work aimed at identifying markers for the CMD2 locus and resistance to CGM but substantial recombination between the markers and the traits diminished their value. Mutliple QTLs for complex traits such as drought resistance have not been available because of the poor understanding of phenotypic variation for sources or resistance on one hand, and poor predicting capabilities of the markers so far identified on the other.

A fifth application, not mentioned by Xu and Crouch (2008), can be to identify and exclude monogenic resistance in a selection scheme to accumulate multiple minor genes with additive effects. At the end of the process, when the target resistance level is reached, the single major gene can be crossed into the population or individual hybrid, to combine both minor and major gene resistance, theoretically for enhanced long-term stability of resistance. For example, this strategy could be followed for combining the single-gene source of resistance to the mosaic disease (CMD2), with resistance from minor genes.

Most successful applications of MAS have been constrained to simple, monogenic traits (Heffner et al. 2009), specifically through the accelerated back-cross introgression of major genes (Xu and Crouch in 2008; Holland 2004). This application of molecular markers, however, faces the problems stated above in the case of cassava.

As stated by Dekkers and Hospital (2007) more than a decade ago, MAS can be readily used for traits with relatively simple inheritance (few major genes with relatively low effect from the environmental conditions). However, for complex traits, with low heritability values that are strongly affected by the environment, MAS faces the same problems that conventional breeders do and, therefore, has had limited impact. One important change in the last decade has been the remarkable reduction of genotyping costs, which allows mass screening at early stages of selection, not previously cost effective. Heffner et al. (2009) identified two weaknesses in MAS that explains their limited success in improving complex traits: (a) the mapping populations used in most QTL studies do not readily translate to breeding applications; and (b) the inadequacy of the statistical methods used to identify target loci and implement MAS for the improvement of polygenic traits. In addition to these problems, MAS has typically concentrated on the improvement of a single trait, whereas breeders need to develop final products that combine a diversity of desirable traits (Xu and Crouch 2008; Nakaya and Isobe 2012). Cassava is not an exception and large efforts have been targeted at disease resistance (as the literature reported above demonstrates) but MAS for more complex traits has yet to find ready applications (Blair et al. 2007). Perhaps in the future, MAS could be used concurrently for the selection of both simple traits with relatively high heritability, as well as more complex traits, but the efficiency of the methodology would have first to be tested and validated.

In the case of cassava, a large amount of molecular information has been reported since the publication of the first molecular map nearly two decades ago (Fregene et al. 1997). The number of articles related to molecular markers is vastly larger than those describing quantitative or Mendelian genetics. Considerable efforts have been invested in identifying molecular markers related to resistance to CMD (Akano et al. 2002; Fregene et al. 2000, 2004; Lokko et al. 2005; Egesi et al. 2006; Okogbenin et al. 2007; Rabbi et al. 2014); bacterial blight (Jorge et al. 2000, 2001; López et al. 2003, 2007; Wydra et al. 2004); CBSD (Kulembeka 2011; Kulembeka et al. 2012); and other diseases (Akinbo et al 2007; Tomkins et al. 2004). Molecular work has also been aimed at identifying markers for tolerance to abiotic stresses (Lokko et al. 2007; Sakurai et al. 2007), as well as in relation to PPD (Cortés et al. 2002).

Many articles describing the applications of molecular markers to cassava breeding have been written over the years (Anderson et al. 2004; Blair et al. 2007; Chavarriaga-Aguirre et al. 1998; Daniell et al. 2008; Ferguson et al. 2011; López et al. 2004, 2005; Fregene et al. 2006; Marmey et al. 1993) and different types of molecular maps have been constructed (Chen et al. 2010; Ferguson et al. 2012; Fregene et al. 2001; ICGMC  2014; Kunkeaw et al. 2010, 2011; Mba et al. 2001; Okogbenin et al. 2006; Rabbi et al. 2012; Sraphet et al. 2011; Tangphatsornruang et al. 2008). Molecular markers research has also explored areas related to nutrition such as cyanogenic glucosides (Kizito et al. 2007) and carotenoids content in the roots (Fortes Ferreira et al. 2008; Marín Colorado et al. 2009; Morillo-C et al. 2011; Welsch et al. 2010). Plant architecture, early bulking and root yields have been linked to different types of markers (Okogbenin and Fregene 2002, 2003; Okogbenin et al.  2008; Boonchanawiwat et al. 2011) as well.

In summary, cassava has witnessed the same evolution of molecular markers in relation to genetic improvement observed for other crops, although lagging well behind crops with a much higher commercial breeding investment, such as maize or soybeans. Earlier markers such as random amplified polymorphisms, restriction length polymorphisms, and amplified fragment length polymorphism have been gradually replaced by simple sequence repeat (SSR) markers and single nucleotide polymorphisms (SNPs). Expressed sequence tags (ESTs) have also been developed from complementary DNA (cDNA) libraries. As of 2011, 80,631 cassava ESTs have been deposited in GenBank (Ferguson et al. 2011). A sub-set of nearly 60,000 of these, filtered on quality, has been compiled into the HarvEST:Cassava database (http://harvest.ucr.edu). The RIKEN group has developed an integrated cassava functional genomics platform in collaboration with CIAT and Mahidol University (Thailand) that includes: (1) full-length cassava cDNA resources and ESTs using high-throughput sequencing; (2) a cassava oligoarray containing more than 30,000 genes; (3) an integrative cassava database of international standards; and (4) a transformation system for cassava cultivars (Utsumi et al. 2011). The cassava genome has been recently sequenced (Prochnik et al. 2011). Consolidated information can be found at http://cassavabase.org/cview/map.pl?map_id=3.

In spite of the large efforts and financial investments in identifying molecular markers to make cassava genetic enhancement more efficient through MAS, the practical application has been negligible (de Oliveira et al. 2012). To date, the only example of molecular markers successfully applied for selection purposes in cassava has been for resistance to CMD in a location where the disease is not present (Okogbenin et al. 2007). In addition to the reasons explaining the limited impact of MAS across crops already mentioned by Heffner et al. (2009) and Xu and Crouch (2008), there are other factors that are specific to cassava and other clonal crops described below.

Most QTL reports to date have been based on segregating populations generated, typically, from two inbred lines (Xu and Crouch 2008). However, in the case of cassava (as well as in other crops such as potato and sweetpotato), heterozygous progenitors have always been used in the mapping populations (e.g., pseudo-F_2_ populations derived from the cross of two heterozygous progenitors). This has been often mentioned in the literature as another explanation for the limited impact of MAS in cassava. The use of heterozygous progenitors results in the need to develop two maps per mapping population. This problem has been recently reduced through the use of new software such as JoinMap 4.1 (Van Ooijen 2011).

A large number of SSR markers were developed independently by different research groups. Therefore, several SSR primer pairs have different names for the same SSR. These duplications, therefore, had to be identified using the recently developed cassava genome assembly as a reference. This allowed the consolidation of a total of 2146 non-redundant SSRs (Ferguson et al. 2011). This curated data set should now become more useful.

Epistasis complicates the identification of associations between markers and phenotypic performance. Complex traits, such as root yield, involve important epistatic effects (Table 2) and this is another reason why MAS has failed to help conventional breeding improve complex characteristics. Understanding epistatic effects is likely to depend on the use of specific genetic materials such as near isogenic lines (NILs) to reduce the complexity of genetic effects, populations of large sample sizes, and suitable statistical methods (Xu and Crouch 2008). However, NILs cannot be currently developed in cassava for reasons explained later in this article.

There has been an ever-changing set of MAS technologies but relatively little actual benefit derived from their use for applied cassava breeding. Availability of high-density maps and falling costs of genotyping will open new opportunities. However, breeders are well advised to analyze efficiency and efficacy of various options, whether cutting edge or more conventional technologies. To date, there has been insufficient follow-through from the identification of markers to application in cassava breeding. Often gene tagging in cassava has been a component of a relatively short-term project, and did not receive the necessary follow-up in implementation. A prioritization of traits in relation to MAS is, therefore, fundamental for practical results: molecular breeding should have a clear advantage over field-based selection and must be feasible in the short to medium term (Blair et al. 2007). A molecular marker that is not used as anticipated may provide insights into genetics and breeding, but can also be an unacceptable waste of resources. This is particularly true in cassava because of the limited resources allocated for research in this crop and the weakness of the national agriculture research institutions of many of the countries where cassava is grown. With the advent of new marker technologies that allow a considerable increase in the density of markers and a significant reduction in the costs per marker, it may be worth revisiting some of the earlier QTL studies. High-throughput SNP genotyping, a sequenced cassava genome, the perspectives of GS to accommodate selection for quantitative traits, and an understanding that MAS is best applied to simple traits such as disease resistance are important developments which should contribute to greater impact of marker technologies in cassava genetic improvement.

Finally, the interaction between QTLs and the environment has the same confounding and negative effect for MAS as genotype-by-environment interaction in conventional breeding. To evaluate QTL by environment interaction, precision phenotyping across multiple locations or environments is required.

Obtaining reliable phenotypic data for complex traits is especially difficult and is often the biggest bottleneck to the eventual application of MAS (Blair et al. 2007; Ceballos et al. 2012). For the proper phenotypic evaluation of genotypes in a mapping population, they need to be multiplied clonally. As is also the case for conventional breeding, the production of planting materials for multi-location trials may require 4–5 years because of the low multiplication ratio in cassava. The chronic problems related to the availability of adequate amounts of planting material result in limitations to the reliability of phenotypic data (e.g., inadequate number of replications, small plot size, reduced number of locations, etc.). There are, however, rapid multiplication systems that can be applied to reduce the time frame for phenotyping, in high priority studies where adequate resources are available.

Cassava’s long growth cycle may be a source of experimental errors in phenotyping of mapping populations. Since cassava does not reach a clearly defined mature state, plants can be harvested at different ages, sometimes with contrasting results. For example, root DMC can vary drastically with the arrival of the rains after a dry period. Variation in the quality of planting material that results from variation in the biotic or abiotic environments across a long growing season is another source of experimental error.

## The potential of genomic selection for cassava improvement

Genomic selection simultaneously estimates many loci, haplotypes, or marker effects across the entire genome to estimate genomic estimated breeding values or GEBV (Meuwissen et al. 2001; Heffner et al. 2009), which can be used to accelerate recurrent selection schemes. It offers several advantages and overcomes key problems of MAS based on QTLs. Although originally developed and successfully used in animal breeding (Dekkers 2007), simulated data suggest that it could have a great impact in crop breeding as well (Meuwissen et al. 2001; Heslot et al. 2012), including cassava (de Olviera et al. 2012) and crops like oil palm (Wong and Bernardo 2008) with which cassava shares many characteristics from the breeding viewpoint.

The GS models presented by Heslot et al. (2012) included different plant species: wheat (*Triticum aestivum* L.), barley (*Hordeum vulgare* L.), *Arabidopsis thaliana* (L.) Heynh. and maize inbred (*Zeamays* L.) datasets. In all these cases, the progenitors are inbred homozygous lines. In cassava, the prospects for using GS have been highlighted by de Olviera et al. (2012). GS offers many advantages over previous molecular technologies. Rather than using mapping populations, the identification of relevant markers (SNPs) is done on training populations–breeding populations similar to those used by cassava breeders. GS does not require prior knowledge of QTL positions in linkage maps. A key advantage is that several different traits can be improved simultaneously through a selection index, also similar to those breeders use. GS efficiently analyzes jointly all markers on a population including loci with small effects (provided that there is a dense, genome wide marker coverage). Different articles emphasize that GS would maximize genetic gains by unit of time (de Olviera et al. 2012; Heffner et al. 2009).

The advantage of reducing cycle time for carotenoids content in cassava has been demonstrated (Ceballos et al. 2013). In fact, this rapid progress to increase carotenoids content made in cassava can be used to illustrate the potential that GS has to offer. Mackay and Caligari (2000) have also highlighted the relevance of reducing the length of each recurrent cycle in simulation studies.

Like conventional breeding and traditional MAS, GS has serious limitations for the selection of low narrow-sense heritability traits because of their low additive genetic effects in relation to the phenotypic variance (Nakaya and Isobe 2012). Genotype-by-environment interactions also affect the precision of GS estimates as well as the relatedness among genotypes (Ly et al. 2013). GS is not expected to be efficient in improving traits in which non-additive genetic effects (dominance and epistasis) are prevalent. In traditional MAS, the dominance effects can be exploited because interval mapping and linkage disequilibrium mapping can predict the dominant effect of QTLs. However, improving modeling algorithms could overcome current problems that GS has regarding dominance effects. By contrast, the consideration of epistasis in GS is more challenging. Epistasis demands vast amounts of computational resources for its identification, and the consideration of epistatic effects in GS is an issue that needs to be addressed in the future (Nakaya and Isobe 2012).

There is an ongoing project (NextGen Cassava Project) to test GS in cassava lead by Cornell University with field work in Uganda and Nigeria (Fessenden 2014). This study can shed some light on the potential usefulness of GS in other clonally propagated crops as well. It is also breaking ground as it analyzes the potential of GS for crop breeding practices with heterozygous progenitors, in which genetic variances are partitioned in a different way compared with crops with inbred progenitors such as those described by Heslot et al. (2012). As stated in the introduction of this article, cassava breeding offers unique opportunities and problems. It will be very informative to see what are the actual gains achieved through GS and what traits it can clearly help to improve in cassava. As illustrated in Table 2, additive variances for resistance to CMD are very important and should, therefore, efficiently be exploited by GS. Perhaps the resistance from CMD2 (Rabbi et al. 2014) can be combined with quantitative sources or resistance that are likely to be present. It is not clear, however, what will be the response for DMC and FRY because of the prevalence of non-additive genetic effects (also illustrated by data presented in Table 2).

## Reproductive biology of cassava

Cassava’s reproductive biology has important implications for both conventional and molecular breeding schemes. The species is diclinous and monoecious: either female (pistillate) or male (staminate) flowers are produced in inflorescences (racemes or panicles) within the same plant (like maize). Pistillate flowers occupy the lower portion of the inflorescence and open 10–14 days before the male flowers which are located toward the apex on the same inflorescence. Inflorescences always develop at the apex of the stem. Sprouting of the buds below the inflorescence allows further growth of the plant. Therefore, almost every flowering event results in branching, or sometimes called “forking”, although non-terminal inflorescenses have been observed rarely (personal observation of authors). Some genotypes flower frequently (3–5 times during a growing cycle) and others flower little or late, or not at all. Erect, late-branching types are frequently preferred by farmers because this plant architecture facilitates cultural practices and results in good production of vegetative planting material and transport and storage is easier. On the other hand, in some systems, early branching is preferred because these varieties close the canopy more quickly and aid in weed control. Synchronization of flowering for planned crosses can be a challenge because some clones flower relatively early at 4 or 5 months after planting whereas others flower only after 10 months. The scarcity of flowers in erect, late-branching types complicates matters further. Because of this and the time required for the seed to mature, it takes generally more than a year to obtain seeds of a planned cross (Ceballos et al. 2012).

The flowering biology of cassava may be challenging for the effectiveness of GS as one of the advantages that is often highlighted is the maximized genetic gains by unit of time (de Olviera et al. 2012). However, if selected genotypes are late to flower, then shortening the duration of each cycle may be difficult. Fortunately, in many cases, genotypes will flower more than once, therefore, allowing crosses with other materials flowering at different times. When CIAT conducted self-pollinations to introduce inbreeding, phenotypes that flowered early and profusely were predominantly selected. This may be an undesirable result of GS in cassava if too much emphasis is placed on a botanical-seed-to-botanical-seed cycle of 12 months. It may lead to producing materials that branch early and this plant architecture is often rejected by farmers. Erect plant architecture would also be necessary for the more mechanized operations envisioned for the future of cassava, especially in Asia and Latin America, but also growing in Africa. Erect plant architecture allows the harvest of longer stems (e.g., 1.5–2.0 m) which have been observed to withstand better lengthy storage periods. This characteristic may prove crucial for the adaptation to the increased uncertainties for the arrival of the rains due to climate change (Ceballos et al. 2011). “Induction of flowering” section below covers options to overcome some of the constraints of cassava’s flowering system for accelerated breeding.

## Inbreeding cassava

Another potentially game-changing ongoing project in cassava aims at the development of a protocol for the production of doubled haploids. Inbreeding in cassava is desirable for the reasons explained below. However, production of homozygous lines in cassava through successive self-pollinations would require up to 12–15 years (if a 2-year cycle is applied) and, as explained above, can potentially encourage the development of early flowering types which are undesirable for many production systems worldwide. Inbreeding offers the following advantages.

### Reduction of genetic load

Inbreeding exposes undesirable recessive alleles whose frequency is moderate to high in heterozygous populations and allows for the elimination of genotypes carrying them.

### Discovery of useful recessive traits

Recessive traits may be desirable and they would be detected by inbreeding. Examples have already been reported for many crops in the literature and are gradually emerging for cassava as well (Ceballos et al. 2007, 2008).

### Implementing the back-cross scheme

The deployment and impact of desirable traits, such as resistance to diseases and pests or special starch quality traits, are slow and limited because their introgression requires breeding for a new variety de novo. Back-crossing is a highly successful breeding scheme used in many crops (Allard 1960; Xu and Crouch 2008), but it cannot be applied to cassava because of the heterozygous nature of the progenitors currently used. If successful cassava clones were derived from inbred progenitors, then the process of trait introgression would be greatly facilitated. This would imply that successful hybrids could be further improved step by step through trait introgression. The current alternative is to cross the source of the new trait with elite breeding materials and then start the long and expensive process of developing an entirely new (hopefully) outstanding hybrid.

### Facilitated germplasm exchange and conservation

If inbred progenitors were available, their conservation and exchange could be through true-breeding botanical seed. Current conservation and exchange operations handle germplasm in vitro which tends to be more restrictive from a quarantine perspective in many countries, as well as costly and slow.

### Development of superior hybrids by design

Hybrid vigor (e.g., non-additive genetic effects) can be progressively improved, but only through reciprocal recurrent selection (RRS) methods (Hallauer and Miranda Fo 1981) or through inbred line development within heterotic groups. Improving heterosis would be slow if no inbreeding was employed. The power that inbred progenitors have had in the maize industry can be appreciated in data presented by Troyer in 2006. About 50–60 % of the gains in maize over the last century are due to genetic improvement (Duvick 1999). Experimental data have demonstrated that heterosis plays a very important role in cassava performance as well (Cach et al. 2005, 2006; Calle et al. 2005; Jaramillo et al. 2005; Pérez et al. 2005a, b). Inbreeding depression, the opposite phenomena to heterosis, has also been found to be significant in cassava, but not high enough to preclude homozygous genotypes producing the few seeds required from them as progenitors for a breeding project (Rojas-C et al. 2009). Moreover, the use of inbred progenitors offers the chance to maintain favorable gene combinations at different loci controlling the small, but critically relevant, non-additive genetic effects as demonstrated in the case of maize (Crow 2000).

### Facilitated maintenance of superior clones

Outstanding hybrids are multiplied and maintained vegetatively. Propagative material (woody stems) eventually gets contaminated by pathogens and other organisms if maintained in the field. If hybrids were produced from inbred progenitors, their planting material could be easily “cleaned” by making the same cross again, as compared to costly and slow tissue culture protocols.

### Facilitated conventional and molecular genetic studies

The availability of homozygous progenitors would facilitate greatly the logistics of genetic studies (both conventional and molecular). Reports in this regard have been published (Gallais and Bordes 2007; Tuvesson et al. 2007).

### Shortening the length of breeding cycles

Currently, elite heterozygous progenitors are crossed among themselves to produce full-sib families. Each F_1_ seed represents a unique genotype. The multiplication rate in cassava is low (one plant produces on average only seven to ten cuttings). It takes 4–5 years to have enough planting material for replicated, bordered-plot multi-location trials. If parents were inbred, multiple pollinations between the same progenitors would yield genetically identical F_1_ hybrids. Therefore, the starting point would not be one but, for example, 30 plants (as currently required for preliminary yield trials) and this would shorten the evaluation cycle by 2 years.

### A more dynamic and efficient breeding method

As traditional landraces are replaced by improved varieties, especially when accompanied by better agronomy, productivity and stability of production increase significantly. Thailand and Southern Brazil are prime examples. Such replacement can be very rapid, but after this initial change, the development and adoption of newer improved varieties tend to occur more slowly. For example, about 93 % of the one million hectares of cassava in Thailand in 2007 was planted with varieties released before 1994. A similar situation occurs in China and southern Brazil, with a prevalence of varieties released in the early 1990s. Genetic gains from traditional breeding systems level off and it becomes increasingly difficult to develop and identify germplasm superior to the varieties already available, unless there is some other major driving factor, such as a new disease or market demand for a new starch trait.

An inbred–hybrid system may offer the potential of a more dynamic and efficient breeding system to move off the plateau of first-generation hybrids that have been widely adopted in some countries. A doubled haploid protocol should be the most efficient way to produce inbreds for such a system. Production of doubled haploids is sought through different strategies based on anther and microspore culture (androgenesis), ovary and ovule culture (gynogenesis) and wide crosses with *Ricinus communis* or irradiated pollen (parthenogenesis). Progress inducing cell division in gametic tissue has been considerable during the past few years (Perera et al. 2012, 2013) and research is now focusing on regenerating plants.

## Induction of flowering

Because of the reproductive biology of cassava, matching flowering dates of genotypes to be cross-pollinated may be a constraint for breeding. Some clones start flowering early, others relatively late, and some clones may never flower. Sometimes, a planned pollination between two clones cannot be done because of the lack of synchronization of flowering dates. To produce the number and types of recombinant seed required for cassava breeding, the same genotype may need to be cloned and planted at different times during the year. Also, up to several thousand plants (depending on size of crossing program) need to be inspected day after day throughout the year, in order to identify the availability of receptive flowers. Male flowers usually outnumber female flowers. The number of female flowers available for pollination is frequently a limiting factor for mass production of hybrid seeds.

A major constraint for cassava breeding, therefore, lies in the amounts of labor and time required for producing the seed required. It is estimated that about 12–15 years may be needed for the transfer of a useful gene from one clone to another, including the complete breeding process of multi-location, multi-year testing to assure end-user acceptance, and multiplication for distribution to farmers. If cassava is going to meet its potential as one of the leading crops in tropical countries, satisfy the growing needs for food, particularly in Africa where it is a staple crop, and become a source of higher income for farmers, a system to speed up the breeding process is urgently needed. Since flowering in cassava is under genetic control (phenotypically expressed as erect versus branching types), it should be possible to stimulate flowering by the exogenous application of phyto-hormones or through grafting approaches. Research is ongoing through the NextGen project between Cornell University and IITA to induce flowering. At CIAT, grafting of non-flowering stems into a profuse early branching understock failed to induce flowering the season the graft was made, but there seems to be a carryover effect when the grafted branch is used as planting material for the following season. A protocol for the induction of flowering may have already been attained at Guangxi Academy of Subtropical Sciences (Information presented by H.Ceballos during the NextGen meeting held in Uganda in February, 2015).

## Exploration of genetic resources and gene/trait discovery

At CIAT, which holds the world’s largest and most genetically diverse collection of cassava landraces, many accessions have been evaluated in diverse environments for basic agronomic traits, resistance to common pests and diseases, and several root quality traits. However, much more can be done for trait discovery and transfer of improved varieties for farmers. An aggressive and comprehensive phenotypic characterization of the crop is yet to be done. Important traits such as amylose-free starch remained hidden for decades until its discovery in 2006. In the case of yams, a source of amylose-free starch was reported only few years ago (Pérez et al. 2011). We still do not fully understand what cassava and other root and tuber crops have to offer. Further evaluation of landraces in different environments for the identification of new sources of resistance/tolerance to abiotic and biotic stresses needs to be carried out. Systematic evaluation in the laboratory for additional nutritional and other post-harvest quality traits in roots and foliage also needs to be conducted. Only 10 % of the cassava germplasm collection has been analyzed for root mineral contents (such as Fe and Zn) at CIAT (Chávez et al. 2005). The more comprehensive the evaluation of the germplasm collections, the more quickly breeders will be able to source and utilize new traits as they are prioritized by growers, processors and consumers.

The ontology of storage roots is among the traits that are poorly understood in cassava. Since roots are the most important commercial product of cassava, this vacuum in knowledge is particularly limiting. Alternatives such as ground penetrating radar may allow a non-destructive monitoring of root development in situ and should be explored. This technology would be particularly relevant in breeding for early bulking, a research objective that is particularly relevant for Africa (Okogbenin and Fregene 2002). CIAT is currently developing a non-destructive methodology to monitor root development through time. The protocol would be similar to aeroponic systems developed for other crops. The initial phase, however, relies on growing the plants in sand which is washed away every time the researcher needs access to the root system for evaluation and/or sampling of tissue. A system for sampling root tissue without the induction of physiological deterioration has been developed already (García et al. 2013). This protocol could be useful, for example, for analyzing gene expression profiles during starch accumulation in storage roots, biosynthesis of starch or carotenoids, development of the starch granules within amyloplasts, etc.

One highly productive approach to the search for new traits has been self-pollination. Although only about 10 % of the CIAT germplasm collection has been self-pollinated, the usefulness of this approach was demonstrated by the discovery of the amylose-free mutation (Ceballos et al. 2007). Molecular tools such as TILLING could be used for the identification of germplasm carrying useful traits (Chen and Dubcovsky 2012; Cooper et al. 2013; Marroni et al. 2011; Tsai et al. 2011).

It is important that every cassava researcher becomes very familiar with the crop. It is only after many hours in the field that the breeder can understand how this crop develops and reacts to different environmental conditions and biotic pressures, and how farmers and consumers perceive and prioritize different traits. Crops like maize, rice, soybean and potatoes have benefited from a century of research in developed countries. The phenotypic variation in these crops had been explored and documented well before the advent of modern molecular tools. Cassava research, on the other hand, is lagging regarding development of genetic stocks and identification of sources for different desirable traits. Molecular markers cannot be linked to traits that are not known to exist. Young scientists may be encouraged to think that “advanced technologies” are more likely to have a positive impact on the crop than they actually offer. Advanced technologies can only stand on top of the foundation of “basic technologies”, which in the case of cassava, are still very weak and incomplete. Many academic programs in Africa rightly encourage students conducting research in cassava to get acquainted, or work directly, with molecular markers technologies. But the emphasis and the current priority should be to also include a strong and broad foundation of applied research based on farmer and other users’ needs. If molecular markers are to have an impact in cassava breeding, every cassava researcher involved in the genetic improvement of the crop would ideally have a clear understanding and experience in conventional breeding as well, so they can properly assess the relative advantages and problems that basic and advanced approaches have. In addition, highly integrated teams of field and molecular breeders need to be built to optimize success rate in genetic improvement.

## Advantages and constraints of the different breeding tools and methods

The foregoing discussion lays out a range of options that can potentially contribute to accelerating genetic gain in cassava. The current section will assess further the merits and challenges of these options in defining an optimal strategy for the genetic improvement of cassava, with lessons for other clonally propagated crops. Fortunately, there are many ongoing activities aiming to overcome some of the problems that cassava breeding presents and to take full advantage of the potential of this remarkable crop. In many cases, however, only preliminary results can be mentioned and not always from published literature.

A reliable method for the induction of flowering would have a large impact because it would benefit all cassava breeding programs. It would also have a synergistic effect on every other technology. For example, the induction of flowering would be highly desirable for GS as well as for the production of inbred progenitors through successive self-pollinations. The production of mapping populations would be facilitated as well. Countries located at subtropical latitudes (e.g., China and Argentina) have chronic problems to produce botanical seed because of the short growing season. Induction of flowering would allow these countries to make their own crosses in locally planted crossing nurseries. The large efforts by the NextGen project and the promising results from Guangxi Academy of Subtropical Agriculture Sciences justify the current optimism that within few years flowering will be routinely induced in cassava crossing blocks by different breeding programs.

The production of inbred progenitors is an appealing possibility for breeders. The availability of inbred progenitors would have a beneficial effect on MAS as well as in conventional breeding (Gallais and Bordes 2007). Back-crossing would be feasible (allowing MAS to have the impact in cassava that has been reported in other crops) and exploitation of heterosis would be by more systematic design. As mentioned above, outstanding commercial maize hybrids depend on non-additive genetic effects (both dominance and epistasis). The only way to preserve the allelic combinations responsible for this genetic superiority is through the use of inbred progenitors (Crow 2000).

The development of inbred progenitors based on doubled haploid technologies will likely be somewhat genotype-dependent in the early stages. Not every cassava breeding program may be able to implement the technology, at least, initially. It may be envisioned, however, that as inbred lines are derived from outstanding popular clones by the larger breeding projects, these lines can then be shared with other breeding projects which may evaluate them through the hybrid progenies they produce when crossed with local germplasm. Available evidence indicates that inbreeding depression would not prevent the capacity of inbred genotypes to flower and produce seeds. In fact, inbreeding seems to encourage flowering and seed set in ongoing research in Uganda (R.S. Kawuki, personal observation).

GS is very promising as a proof of concept but its actual potential is yet to be demonstrated. It is still expensive and, therefore, restricted to a few programs. However, as more information is developed, assuming that the cost of genotyping will keep going down and that the protocols for flowering induction and doubled haploids production are developed, it may become an important approach for cassava genetic improvement in the future, at least for relatively large breeding programs. Genotype by environment interaction affects the precision in GS in cassava (Ly et al. 2013) and still needs to be addressed better. GS could be applied in the enhancement of reciprocal populations once heterotic patterns have been identified or created. MAS will continue to have specific applications as before. It may be useful for identifying recessive traits. It would be a key technology for accelerated back-crossing schemes, once inbred progenitors can be generated.

## The future of cassava breeding

**Fig. 1:**
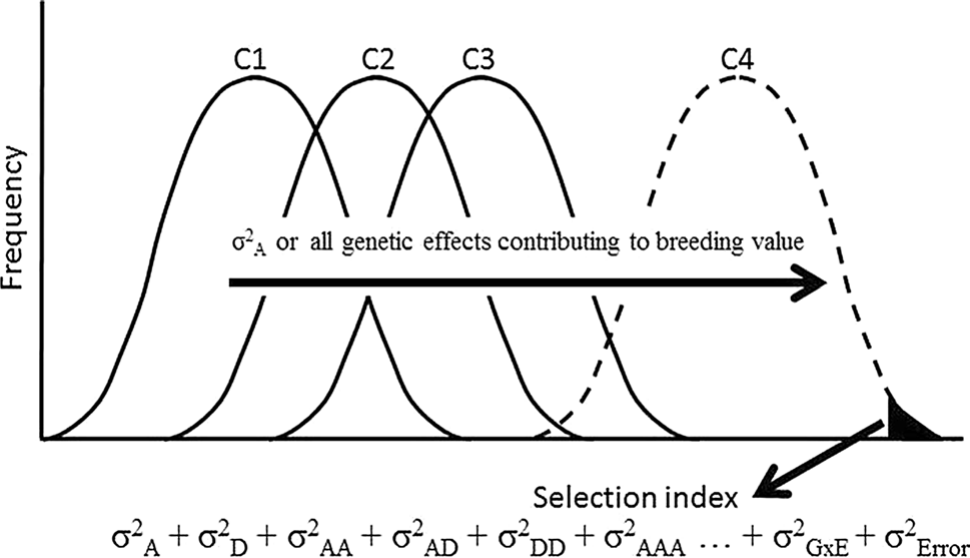
Illustration of a truncated phenotypic recurrent selection scheme such as the one currently used in cassava breeding. C1, C2, C3 and C4 are the successive cycles of selection. Shifts in allelic frequencies gradually occur in the different versions of the population represented by the successive cycles. This genetic progress is achieved mostly exploiting additive genetic effects. The selection of a successful clone, however, is affected by all genetic effects as well as experimental errors and the ever confounding effect of genotype-by-environment interaction

Figure 1 illustrates the typical response of conventional recurrent selection methods as well as the expected impact of GS. The process relies on exploitation of breeding values which are linked to additive genetic effects. However, farmers grow single outstanding clones, not populations. Shifting the average performance of the population is not the end of the breeding process because successful hybrids from the breeding populations still need to be identified. Several years ago, Good (1990) reviewed the use of recurrent selection in commercial maize seed companies in the USA. Most companies used recurrent selection, but on average they invested only about 10 % of their breeding resources in this strategy. The rest of the investment was toward development of inbred lines and identification of superior hybrids. This may provide an insight into the relative costs (or envisioned benefits) involved in population performance versus developing and identifying an outstanding product that the farmer will grow.

The most challenging problem in cassava breeding is probably how to identify the best genotype out of the billions that two (heterozygous) progenitors could hypothetically produce. Certainly, there is a limit to the number of genotypes that can be used to represent a given full-sib family and this is a major limitation that GS as well as conventional cassava breeding face. Perhaps thousands of crosses between Thai clones Rayong 1 and Rayong 90 have been made, but only one commercially successful superior genotype has been selected from these crosses so far: the widely grown variety KU50. GS can efficiently predict the best progenitors to be recombined to generate a new and improved version of the breeding population because of its capacity to identify desirable individuals based on their GEBV. It is the selection of a single outstanding genotype out of hundreds representing the same full-sib family, however, that makes cassava breeding challenging. At CIAT, a truncated selection is made based on a selection index. In this process, complications derived from genotype-by-environment interaction, large experimental errors, the complex and unpredictable effects of dominance and epistasis merge to make the breeders’ task difficult (Fig. 1). As demonstrated above, these non-additive effects are particularly relevant for complex traits such as yield.

**Fig. 2:**
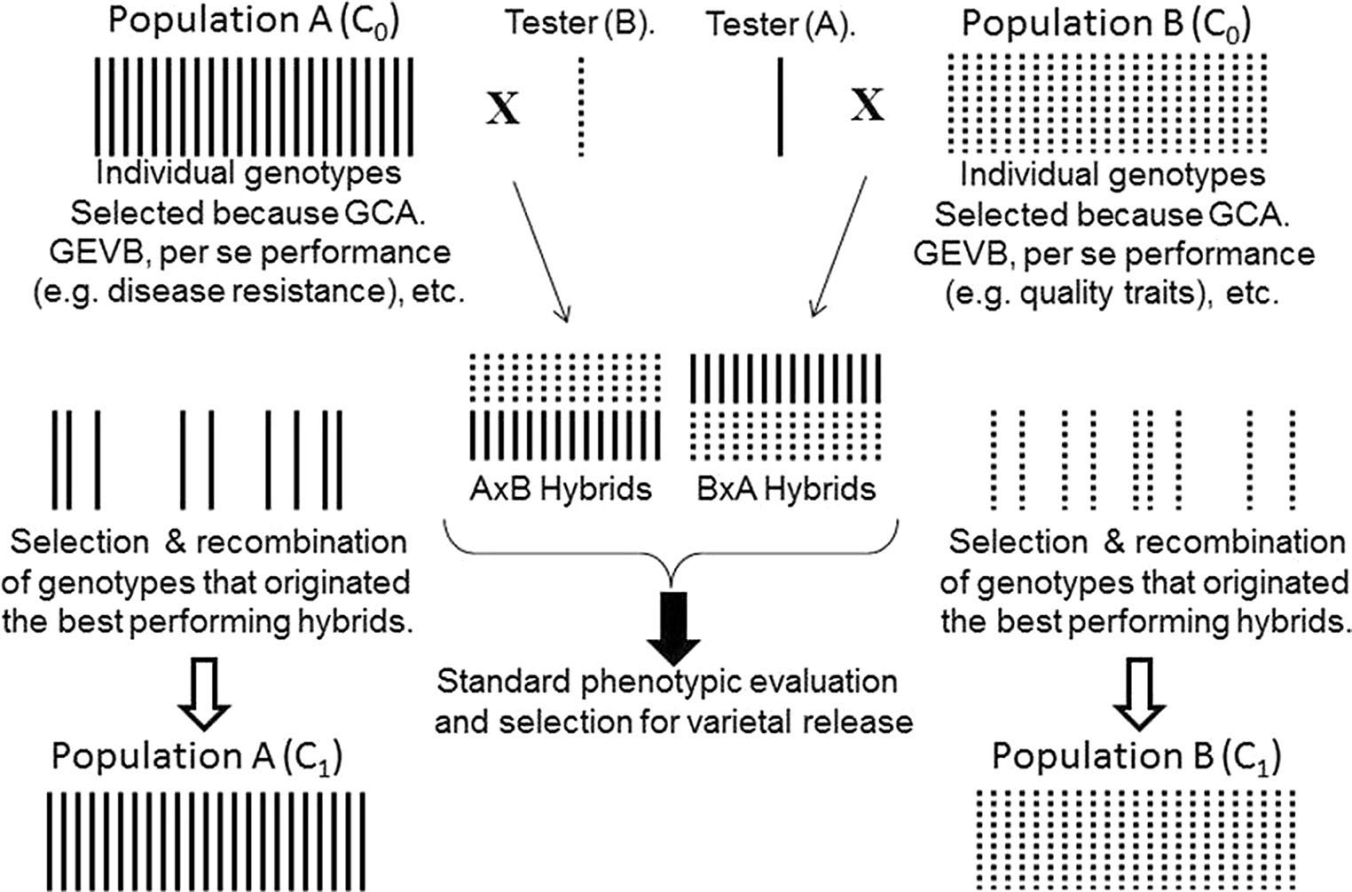
Illustration of a typical scheme of reciprocal recurrent selection of two heterotic populations. Hybrids are made through crosses of selected gentoypes from each population with a tester from the reciprocal population. Progenitors of the best hybrids are combined (within each population) to start a new cycle of selection

Implementing an RRS scheme (Hallauer and Miranda Fo 1981) would improve the cassava breeders’ ability to exploit heterosis. For RRS, at least two heterotic populations would be defined (Fig. 2) based on their SCA. In maize, the improvement over the years of the per se performance of each population has been linked to additive genetic effects (e.g., breeding value). The performance of the crosses between the two reciprocal populations can also be improved over the years. Recombination of selected genotypes to start a new cycle of selection occurs only within each population. It is this particular restriction of the RRS that allows the gradual and consistent exploitation of non-additive genetic effects ($\sigma_{\text{D}}^{ 2} \; + \;\sigma_{\text{AD}}^{ 2} \; + \;\sigma_{\text{DD}}^{ 2} \; + \;\sigma_{\text{ADD}}^{ 2} \; + \;\sigma_{\text{ADD}}^{ 2} \; + \cdots {\text{etc}}.$).

RRS, rather than exploiting all genetic effects simultaneously, can separate (to a certain extent) the improvement of additive and non-additive genetic effects, an advantage that counterbalances its added complexity. The separation of the breeding project in two heterotic groups is the only way to guarantee a gradual improvement of non-additive genetic effects. RRS has been implemented in maize, cotton, eucalyptus, gourd, oil palm, pearl millet, rice, sorghum, and tomato (Bernardo 2014). In vegetable breeding (Knapp formerly of Monsanto Company, personal communication), heterotic populations can be used as pools for different traits. One population, for example, can be the source for defensive traits while the other could provide desirable quality traits to the resulting hybrids.

The implementation of an RRS scheme adds complexity to the overall breeding approach as there are two levels of crosses made: (a) within populations among the progenitors of the best performing hybrids, to start a new cycle of selection; and (b) between populations for evaluation of hybrid performance and identification of those that carry good GCA effects but also expose the “spark” of specific combining ability that distinguish outstanding hybrids from the average.

The identification of heterotic patterns in cassava germplasm is an important goal that is urgently needed because they are the backbone of successful hybrid breeding and of RRS (Melchinger and Gumber 1998). Complementary heterotic patterns are identified in a pair of populations (or individual inbred genotypes) which express high heterosis and consequently high hybrid performance in their cross. Germplasm resources have been widely exploited in the case of maize and clear heterotic patterns have been developed for temperate, subtropical and tropical adaptation (Hallauer and Miranda Fo 1981; Pandey and Gardner 1992; Melchinger and Gumber 1998; Parentoni et al. 2001). It is not possible to predict whether heterotic patterns in cassava can be found among diverse gene pools or if they will have to be created de novo. It is safer to assume that heterotic patterns will probably have to be constructed and the sooner this work begins the better.

Genetic distance has been shown to be of little value as a predictor of heterosis, (Cress 1966; Crossa et al. 1987; Fu et al. 2014; Melchinger 1993; Melchinger and Gumber 1998; Pérez-Velázquez et al. 1995). In fact, preliminary (unpublished) results in cassava indicate that genetic distances fail to predict specific combining ability effects in the three diallel studies conducted at CIAT (Cach et al. 2006; Calle et al. 2005; Jaramillo et al. 2005). Assessment of heterosis, therefore, should focus on diverse accessions that have resulted in the production of superior hybrid clones. For example, progenitors of successful hybrids (such as the widely grown clone KU50 developed in Thailand) can be used as a source of (partially) inbred lines that can eventually lead to an approximation of the gametes that gave rise to that particular hybrid. The availability of inbred lines would facilitate the development of heterotic patterns as all genetic variation is among crosses and there is no within-family variation.

In late 2014, a meeting to discuss the exploitation of heterosis in cassava and other asexually propagated crops was held at CIAT (see Acknowledgments Section). Participants largely agreed that implementing a breeding scheme to exploit heterosis was sound. In fact, RRS is already a reality in the case of sweetpotato. It was also agreed that key steps would be the identification of heterotic groups on one hand, and implementation of some sort of recurrent selection on the other.

Finally, if there is a possibility of producing partially or fully inbred lines, the exploitation of heterosis could be further maximized and the combination of different traits facilitated. The use of inbred progenitors offers advantages already mentioned above. When progenitors are inbred, there is no within-family variation in the cross they generate. This is convenient because the evaluation of hybrids can begin at the preliminary or advanced yield trial stage, reducing almost in half the lengthy current evaluation system (Ceballos et al. 2012). Heterotic responses are also more clearly identified when homozygous progenitors are used. The performance of inbred lines per se represents twice the $\sigma_{\text{A}}^{ 2}$ present in the original F_1_ cross and does not expose any dominance genetic effect (Table 1). Selection among inbred lines within each reciprocal population can, therefore, only act on additive effects ($\sigma_{\text{A}}^{ 2} \; + \;\sigma_{\text{AA}}^{ 2} \; + \;\sigma_{\text{AAA}}^{ 2} + \cdots {\text{etc}} .$). The performance of the inbred lines per se would allow reducing the frequency of undesirable alleles (genetic load), which results in improvement of their breeding value. GS could be used to select for GEVB, and offers clear advantages if the progenitors were homozygous.

**Fig. 3:**
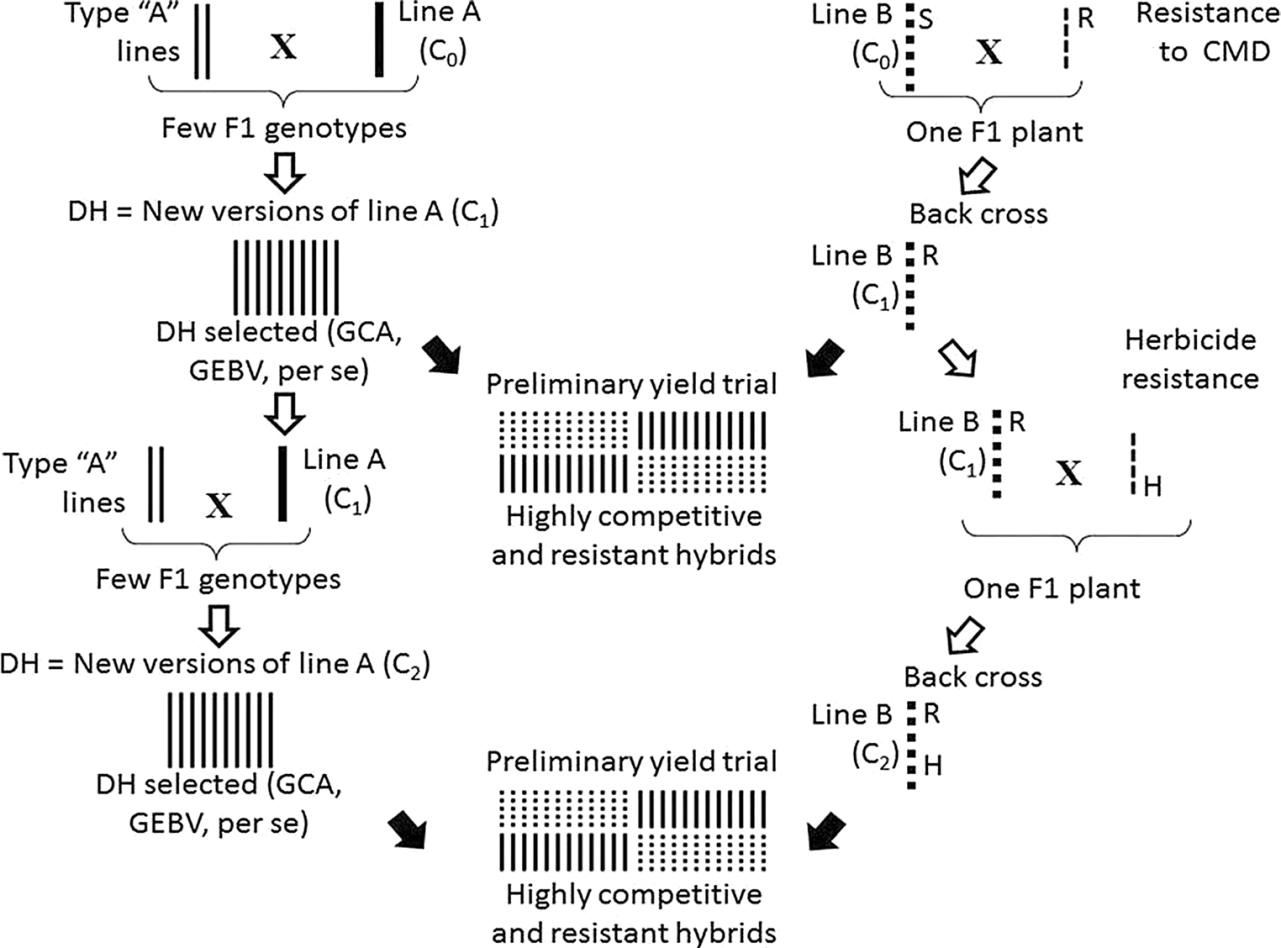
A breeding scheme for cassava based on the use of inbred progenitors from two heterotic populations. Solid *black arrows* indicate the between heterotic group crosses for production and evaluation of experimental hybrids. *White arrows* indicate within-population variation. *Line A* is gradually improved for its heterotic response when crossed with *line B*. On the other hand, *line B* is improved for resistance or quality traits

The performance of the hybrids between the two heterotic populations, on the other hand, would also allow the expression of non-additive genetic effects. As in the case of maize, inbred cassava progenitors would allow for a stepwise exploitation of heterosis. This approach may quickly result in the identification of two inbred lines leading to an outstanding cross such as the venerable maize hybrid B73 x Mo 17 (Nelson et al. 2008). When breeding reaches this stage, there will no longer be an RRS system, but rather a line improvement process (Fig. 3). Hybrids would be improved through directed changes in their progenitors. Line improvement relies on crossing related lines to generate limited genetic variability in search of improvement of quantitative traits (as illustrated for line A in Fig. 3), or else through trait introgression for simple inheritance traits. Inbred lines that produce outstanding hybrids could be improved through accelerated backcross schemes based on MAS (as illustrated for line B in Fig. 3, first for the introgression of resistance to CMD and then for herbicide resistance).

**Fig. 4:**
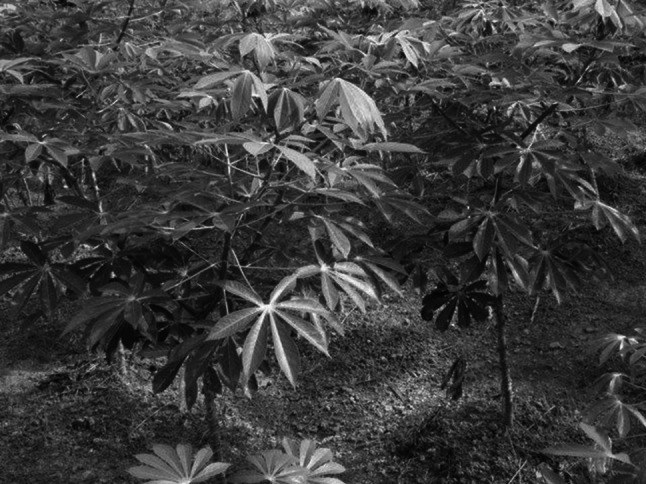
Photograph of S_5_ cassava genotypes produced and grown at CTCRI in Kerala State, India. Notice the uniformity of the two plants shown. These plants come from botanical seed. The uniformity is indicative of the high degree of inbreeding in their parental genotype. Also notice that they have already flowered at least once (as branching has already taken place)

Cassava is unique among many clonally propagated crops because of its diploid status (Wang et al. 2011; Aiemnaka et al. 2012), lack of self-incompatibility and evidence suggesting that inbreeding depression for the production of viable seed would not be a major obstacle (Rojas-C et al. 2009; Fig. 4 presents a photograph of S_5_ genotypes taken at CTCRI in Kerala State in India). These characteristics improve the likelihood that inbreeding and the doubled haploid technology are feasible.

Another less tangible but important advantage of inbreeding is the collaboration that would emerge among the breeding projects. Sharing inbred lines with good general combining ability and known specific combining ability would foster integration among the few cassava breeding programs in the world, very much as Land Grant Universities in the USA and other programs worldwide collaborated in maize breeding during most of the last century (Mikel and Dudley 2006). In cassava, for example, a good inbred line developed for high root yield in Thailand could be combined with another inbred line developed for CMD resistance in Nigeria for the production of outstanding hybrids in West Africa. This kind of international collaboration is very limited nowadays and, therefore, enhancing it would have a large impact. We do not know yet what the potential of heterotic expression will be in cassava. Based on current limited data, hybrids superior for DMC and root yield would be more easily identified if they are from inbred progenitors. As our understanding of heterosis and its relationship with particular portions of the genome (or specific loci) grows, then selection-enhanced heterosis could also be achieved through MAS.

The use of inbred progenitors, however, adds complexity to the breeding system. In addition to the two different types of crosses made in RRS (within populations to start a new breeding cycle and between populations for the evaluation of crosses among the two populations), there is a need to extract doubled haploids from each F_1_ cross within populations (e.g., Line A in Fig. 3). The foreseen evolution of RRS into line improvement implies a major change because the resulting hybrids produced will all be highly competitive. Today, thousands of hybrids need to be evaluated and only a few will show an outstanding performance. The overall efficiency of the process should be improved and the gains per cycle increased. The complexity of a breeding system would be higher but only until a few outstanding pairs of heterotic lines are found. Thereafter, the system becomes easier to handle: inbred line improvement is simpler and more predictable than population improvement and, more importantly, the average performance of experimental hybrids is expected to increase significantly.

The breeding scheme depicted in Fig. 3 provides an illustration of how cassava breeding could evolve to integrate most of the technologies described above. Doubled haploid lines could be developed from the African, Asian and American advanced cassava breeding programs. Sharing these inbred lines would be easy as could be done as botanical seed. As in the case of maize, the exchange of homozygous lines from different gene pools should eventually help in the identification of heterotic patterns.

The current article presents advantages and challenges associated with the implementation of RSS and shifting to the use of inbred progenitors. The experiences achieved in cassava could then be applied to other root and tuber crops. In the near future, it will be highly beneficial to conduct modeling studies (e.g., Shaw and Hood 1985) quantifying advantages and challenges of different approaches. This, together with new information on GS, progresses in the induction of flowering and the availability of a protocol for the production of doubled haploids would provide useful guidelines and a basis for comparing costs and benefits for different approaches toward the genetic improvement of cassava and other root and tuber crops.

### Author contribution statement

Hernán Ceballos prepared the basic manuscript taking advantage of his experience in maize and cassava breeding and knowledge of quantitative genetics in these two crops. Robert S. Kawuki contributed in the writing of the original manuscript using his experience in conventional cassava breeding in Africa and his current participation in the NextGen project to implement genomic selection in cassava. Vernon E. Gracen contributed correcting concepts of heterosis and combining ability in the manuscript and providing ideas on ways to identify heterotic groups. G. Craig Yencho contributed by widening the scope of the manuscript using his experience in sweetpotato and making more accurate the description of some concepts in the original manuscript. Clair H. Hershey together with H. Ceballos wrote the original manuscript using his experience in conventional cassava breeding and conservation and exploitation of genetic resources.
